# Has your smartphone replaced your brain? Construction and validation of the Extended Mind Questionnaire (XMQ)

**DOI:** 10.1371/journal.pone.0202188

**Published:** 2018-08-31

**Authors:** Sari R. R. Nijssen, Gabi Schaap, Geert P. Verheijen

**Affiliations:** Behavioural Science Institute, Radboud University, Nijmegen, The Netherlands; Universita degli Studi di Perugia, ITALY

## Abstract

As digital devices, such as smartphones, are becoming ever more absorbed in the daily lives of adolescents, a major assumption is that they start taking over basic functions of the human mind. A main focus of current debate and research is therefore on investigating adolescents’ use of digital technologies. However, the lack of an instrument measuring the degree to which adolescents offload cognitive and social functions to technology hinders debate and research. This paper tests the reliability and validity of the Extended Mind Questionnaire (XMQ) which measures the degree to which digital technology is used to offload cognitive and social functions. In a first study on young adults (n = 63), we constructed a 12-tem scale, which proved to be highly reliable. A large-scale study on teenagers (n = 947) demonstrated the high structural validity, reliability, and construct and criterion validity of the XMQ. In sum, these studies provide evidence that the XMQ is psychometrically sound and valid, and can be useful in future research on the consequences of digital technology in the daily lives of adolescents.

## Introduction

Mobile devices such as smartphones and tablets play an increasingly significant role in our daily lives. We rely on our digital devices for doing our jobs, maintaining friendships, navigating traffic, or relaxing after work, and our physical and emotional attachment to them has deepened accordingly. One of the most pertinent questions for the 21st century will be how these increasingly intelligent and invasive technologies will affect our minds. Many think digital technologies are fundamentally shaping how we think, process information, and engage in social relationships [1–9]. It is important to develop research methods that inform public debate on how to deal with the innovations that Silicon Valley provides for us. Such methods are currently not sufficiently available.

At the core of the current debate is the notion that today’s digital devices are becoming so thoroughly integrated in our lives, that, for better or for worse, they start taking over basic human functions. The current generation of technology is fundamentally different from earlier innovations in the sense that it is mobile and provides continuous access to limitless networks of knowledge and social contacts [10]. The assumption is that because of these qualities, our devices–and through them the Internet–are becoming a primary form of external memory, taking over this task from the brain [11, 12]. For instance, when people expect to have future access to information, they have lower rates of recall of the information itself and enhanced recall for where to access it instead [10]. Similarly, Barr et al. [1] found that we rely on our smartphone to ‘offload’ cognitively demanding tasks, such as analytical reasoning. Furthermore, we consider our smartphone an extension of ourselves and separation from it heightens state anxiety and impairs executive functioning [2, 13]. The aim of the current study is to develop and validate the Extended Mind Questionnaire (XMQ): a new instrument for measuring the degree to which digital technology is becoming an extension of the adolescent mind. This tool will enable researchers to investigate our relationship with digital devices, and their impact on the cognitive and socioemotional functioning of those growing up with technology.

Adolescents are the front-runners in many of these developments [14]. They are the first generation to feel the full impact in their current and future private and professional lives and education. A main focus of research should therefore be on investigating how adolescents experience the way digital technologies are an extension of their minds. Youth growing up in this environment are often referred to as ‘digital natives’ [15]. Digital natives think and process information in fundamentally different ways than older generations: they are more comfortable with multitasking, reliant on graphics for communication, and thrive on instant gratifications and rewards [16]. While age alone is not a sufficient criterion to label someone a digital native [17], being surrounded by technology throughout development will influence brain functionality [18]. This concerns the educational sector in particular, as it forces changes in teaching styles [19] (but see [20]). Digital technology influences the social domain as well. For instance, 57% of teens have met a new friend online and 70% of social media-using teens feel better connected to their friends’ feelings through social media [21].

Although the idea of a digitally extended mind is a major underlying assumption of research and public debate on the impact of technology on daily life, there is to date no measure of the degree to which we rely on technology or to which technology is embedded in our minds. Research into the causes and consequences of technology use has relied on possibly related measures such as frequency or duration of use (‘screen time’), and on indications of excessive or problematic use such as addiction, constant alertness for notifications, feeling overloaded, or intrusiveness in family life [22–25].

While it is unquestionably valuable to determine whether screen time alone has cognitive and social effects on users, such measures have their limitations: the frequency and duration of device use do not say anything on use in terms of content or (dys-)functionality of the use. This point was recently endorsed in an open letter to the Guardian, in which a large group of scientists argued that screens are not inherently harmful and that the narrow focus on it is hindering any progress in research on the effects of technology on children’s social, emotional, and cognitive development [26]. Similarly, while research on antecedents and consequences of excessive or problematic media use is undoubtedly valuable, this focuses only on the negative side of digital device use, which is not representative of everyday, ‘normal’ use. Moreover, they provide little insight into the prevalence, causes and consequences of the core assumption in the debate: the degree to which we offload cognitive and social functions to our devices.

To fill this gap, the goal of the current study was to construct and validate a scale that assesses the extent to which humans, and digital natives specifically, use digital technology as part of their extended mind. Below, we illustrate how this scale is developed based on the concept of the ‘extended mind’. Subsequently, we assess the scale’s reliability and validity in a large-scale study on adolescents.

### Scale development

To construct a measure of the degree to which individuals experience their devices as digital extensions of their minds, we build upon extended mind and transactive memory literature. In their seminal paper, Clark and Chalmers [27] propose that the human mind is not limited to within the skull: it is an extended cognitive system in which objects in the environment are dynamically embedded and play an active role in driving cognitive processes. This coupling between mind and environment allows objects to aid cognitive processing by enabling the mind to ‘offload’ some of its internal processing onto the environment. For example, when we use a notepad to write down an address, we no longer have to rely on our own scant memory capacity. Thus, digital devices, and through them the Internet, augment or replace functions of the brain, projecting the mind into the physical world [3, 28].

Similarly, we may use the Internet for knowledge we use in daily life. The concept of group or ‘transactive’ memory refers to this function [29–31]. In the non-digital world, groups of cooperating humans usually develop transactive memories These are interpersonal memories consisting of knowledge held by individuals and the memory stores of other group members or devices which can be accessed because the individual knows where the information can be found outside his/her own memory. Thus, an individual can either search his/her own memory for knowledge, or ‘outsource’ knowledge storage and retrieval to external sources, such as other group members, or (note)books, the Internet, and smart phones. It has been argued that the Internet effectively functions as an endless transactive memory, encouraging us to offload memory functions to technology, which suggests we are adapting to new technologies by becoming more symbiotic with our digital devices [9, 10]. Through its capacity, reach, and efficiency, mobile digital technology has an even greater potential to act as an agent of extended mindedness than earlier technologies [32]. Thus, in a very real sense, a smartphone is not simply a tool, but is part of ‘me’ [10, 12].

The concepts of extended mind and transactive memory allow us to conceive of digital technology as an external object that has become coupled with the mind in such a way that it enables us to offload some of our internal cognitive processes onto our smartphones, tablets, and computers, thus effectively becoming an extension of our mind. From this perspective, the increased use of digital technology goes beyond a mere quantitative change in hours spent behind screens: it entails a markedly different, qualitative change to our cognitive functioning. The concept of a digitally extended mind opens up a novel and exciting way of investigating the impact of digital technology on our daily life, for example by assessing whether and how the extent to which we are coupled to digital technology affects how we learn, store and retrieve information, focus our attention, interact with real-life and virtual others, and so on.

To quantify the concept of a digitally extended mind, we created a new scale that assesses the extent to which a person experiences digital media to be a part of their own mind: the Extended Mind Questionnaire (XMQ). This instrument measures the degree of *accessibility*, *endorsement*, and *reliance* a person experiences towards digital media. From the work of Clark and Chalmers [27] and Wegner and colleagues [9, 29, 30], we derived three criteria for objects to be considered part of the extended mind. First, the object should be a constant in a person’s life, and he or she would not take action without consulting the device (labeled ‘reliance’ here). Second, the object needs to be easily accessible in use (‘accessibility’). Third, information from the object should be endorsed automatically (‘endorsement’). A more elaborate explanation of these three aspects of an object that is part of the extended mind can be found in Clark and Chalmers (p. 17 in [27]).

In the development of the XMQ, we followed the roadmap for scale construction that was proposed by Churchill [33] and Gerbing & Anderson [34]. In Study 1, a 28-item questionnaire was developed based on the three criteria for extended mind outlined above. Based on reliability analyses, a reduced 12-tem scale was constructed. In Study 2, the psychometric qualities of the 12-item scale were examined in a large sample of teenagers (12–18 years old). Specifically, we examined the scale’s 1) factor structure and reliability, 2) construct validity, as indicated by discriminant validity, and 3) criterion validity.

Construct validity is the extent to which a scale measures the intended construct. Construct validity assumes the presence of a set of empirical and theoretical laws, i.e., a nomological network, which predicts how different constructs in the network as well as different observations are interconnected [35]. While the XMQ has a strong conceptual basis, due to the recency of the technological advancements and research focus, a nomological network relating digital mind extension to other theoretical or empirical constructs does not yet exist. Therefore, we use discriminant validity to establish construct validity. Discriminant validity tests whether concepts that are not supposed to be related are in fact unrelated. To assess discriminant validity of our new construct, we compare scores on the XMQ scale to one other measure that is currently often used in research on effects of digital media: screen time. Presumably, screen time is related to extended mindedness: the more time one spends with a device, the more opportunity for tendencies to develop to offload functions of the mind to it. In that sense, screen time is most likely a necessary, but not sufficient precursor of extended mindedness. In other words, the two concepts are in all likelihood empirically somewhat related, but not conceptually equal. Therefore, in testing discriminant validity we expect to find only weak (*r* = .10 –.29) to moderate (*r* = .30 –.49) correlations [36] between XMQ scores and screen time.

Second, criterion validity is the degree to which a measure is related to (an) outcome(s). Here we hypothesize that XMQ scores and screen time relate in different ways to other variables that are often presented together in research and public discourse on the effects of digital media: gender, age, time spent with peers, academic performance, problematic social media use, and problematic Internet use.

## Study 1

### Method

#### Sample

63 Dutch undergraduate students (M_age_ = 20.37, 88.9 percent female) participated in exchange for course credit. Participants were recruited via an online participant system and the questionnaire was presented using Qualtrics (http://www.qualtrics.com). All participants gave informed consent prior to their participation. The Institutional Review Board of the Radboud University Faculty of Social Sciences approved the research described in this manuscript under approval number: ECG2012-2505-03.

#### Materials and procedure

Based on the three criteria reliance, accessibility, and endorsement, we created 28 questionnaire items that would probe extended mindedness in both the cognitive and social domain of daily life. The original scale was created in Dutch, but items were translated to English for this report. All English items were back-translated to Dutch by a panel of experts. For this first study, 11 were items created for the reliance criterion (e.g., ‘If I cannot recall something, I immediately reach for my phone, tablet, or computer to look it up’); 9 items were created for the accessibility criterion (e.g., ‘I always have my phone, tablet, or computer within reach’); and 5 items for the automatic endorsement criterion (e.g., ‘I trust information from my phone, tablet, or computer as much as my own memory’). In addition, 3 items were created covering all the criteria in a more general way (e.g., ‘My phone, tablet, or computer is a part of me’). The initial 28-item Dutch questionnaire can be found in S1 Table). The presentation order of the items was randomized for each participant. Participants were instructed to rate to what extent each item described them in their relationship to technology on a 5-point Likert scale ranging from 1 (*Does not describe me at all)* to 5 (*Describes me very well*).

### Results

Reliability analyses were conducted to reduce the total number of items in an iterative process. Items that contributed least to the scale’s internal consistency (i.e., with the lowest inter-item correlations) were removed one at a time until the scale’s reliability could not be increased further (final α = 0.82). This resulted in a 12-item scale that consisted of 4 items in the reliance criterion (items 4, 6, 8, and 11), 4 items in the accessibility criteria (items 5, 9, 10, and 12), 1 item in the automatic endorsement criterion (item 7), and 3 items covering the different criteria in a more general way (items 1, 2, and 3; see Table 1) The final 12-item questionnaire was validated in Study 2.

**Table 1:** XMQ items and factor loadings.

Item	Item wording	Component 1	2
**1**	My smartphone, tablet, or computer is a part of me	**.818**	.121
**2**	I use my smartphone, tablet, or computer as an extension of myself	**.763**	.177
**3**	My smartphone, tablet, or computer often serves as my memory	**.752**	.237
**4**	I am very dependent on my smartphone, tablet, or computer	**.750**	
**5**	Online information is accessible to me at any given time	**.692**	-.149
**6**	If I cannot recall something, I immediately reach for my smartphone, tablet, or computer to look it up	**.688**	-.305
**7**	I trust information from my smartphone, tablet, or computer as much as my own memory	**.663**	.272
**8**	Social media are important to me for contacting my friends	**.649**	-.235
**9**	When I want to get in touch with friends, I immediately reach for my smartphone, tablet, or computer.	**.631**	-.208
**10**	I always have my smartphone, tablet, or computer within reach	**.602**	-.446
**11**	I am very dependent on my smartphone, tablet, or computer to remember important things such as birthdays or appointments	**.558**	.184
**12**	(Reverse score) It makes more sense to me to address someone in person than through social media	.127	.**741**

* (back-translated from English to Dutch by an expert panel)

## Study 2

### Method

#### Sample

The final XMQ scale was submitted to 1066 Dutch high school students as part of the Kandinsky Longitudinal Study (KLS) [37]. The KLS is a research project requested by the school in 2010 in order to help detect youth at risk for social and emotional problems in secondary education. Data collection occurs once every 12 months in one secondary school located in a city in the southeastern Netherlands. Before each wave of data collection, the school signs a letter in which they claim responsibility for the consent procedure. Parents receive a letter describing the procedure of the research at the beginning of the school year and are able to exclude their child from participation. Adolescents provided assent at the beginning of the survey. The Institutional Review Board of the Radboud University Faculty of Social Sciences approved the research described in this manuscript under approval number: ECG2012-2505-03.

The current study included adolescents who were in the 7^th^ to 10^th^ grade in the KLS November 2016 wave. The final sample consisted of 947 respondents (M_age_ = 14.15, 50.3% female); 111 respondents were removed from the dataset for failing to complete the questionnaire and 8 for giving duplicate responses to all items in the scale (including the reverse-coded item).

#### Materials and procedure

In addition to the XMQ items, we administered 3 items assessing screen time with regard to smartphones, tablets, and computers (“How many hours do you spend on your smartphone / tablet / computer on a regular day?”). Participants first responded to these screen time items on a 6-point Likert scale ranging from 1 (*less than one hour a day*) to 6 (*more than four hours a day*). Participants could also indicate if they did not own a smartphone, tablet or computer. Subsequently, the XMQ was presented with the item order randomized. Participants were instructed to rate the extent to which each item described themselves in their relation to technology on a 5-point Likert scale with phrasing provided for the end-points, ranging from 1 (*Does not describe me at all*) to 5 (*Describes me very well*).

Three additional constructs were measured in order to test the predictive validity of XMQ. First, teachers reported the average grade separately for each student, by answering the questions “What is the average grade over all school performances of this student?” Answers were obtained on a scale from one to ten, which is in line with the Dutch grading system. Second, time spent with peers was measured using the question “During weekdays and outside of school hours, how many hours per day do you spend with friends?” Answers ranged from 0 (*None*) to 8 (*More than 7 hours per day*). Third, the severity of problematic Internet use and the severity of problematic social media use was measured with an adapted version of the Compulsive Internet Use Scale (CIUS) [38]. The CIUS in our survey was shortened to six items and included one item for each of the six dimensions of compulsive use (including loss of control, preoccupation, intrapersonal conflicts, interpersonal conflicts, withdrawal symptoms, and coping symptoms; [38]). The CIUS was administered for both Internet use and social media use separately. Items have similar wordings but were rephrased, with the terms *Internet use* changed in *social media use* in the problematic social media use questions. This version of the CUIS is commonly used in Dutch national surveys (e.g., [39]). An example item would be “How often do you feel restless, frustrated, or irritated when you cannot use the Internet?” Answers were given on a 5-point Likert scale, ranging from 1 (*Never*) to 5 (V*ery often*). Reliability was acceptable for both problematic internet use (*α* = 0.82) and problematic social media use (*α* = 0.84).

A computerized survey was administered at school in the classroom. Adolescents sat at a private desk and at least two researchers were present during assessment sessions. In addition, the teacher of each class filled out measurements of academic performance and socio-emotional wellbeing of their students. Instructions were provided verbally and on the laptop at the start of the survey. One session took between 45–60 minutes. Participants filled out the XMQ as part of a larger set of scales, including questions on media use and measures of adjustment that are beyond the scope of the current study.

### Results

#### XMQ dimensional structure

To explore the underlying dimensional structure of the final, 12-item version of the XMQ, the sample was split randomly while keeping age and gender balance equal in both halves of the dataset. With the first random half, an exploratory factor analysis (EFA) with promax rotation was conducted in SPSS Version 23. Results supported one strong factor (Factor 1 eigenvalue = 5.28, 43.99% variance explained) and one medium factor (Factor 2 eigenvalue = 1.18, 9.85% variance explained). The scree plot indicated one factor. Table 1 shows that all items loaded strongly onto the first factor (all factor loadings > .55), with only the reverse-coded item (“It makes more sense to approach someone in real life than on social media”) loading onto the second factor (factor loading = .74). Therefore, the reverse coded item was removed from the analysis in subsequent steps.

To test the dimensional structure, next, a confirmatory factor analysis (CFA) was conducted using the lavaan package [40] in R (http://www.R-project.org) on the second random half of the dataset, fitting a model with one factor. Results indicated that this model provides an acceptable fit to the data, *χ*^*2*^(54) = 200.39, *p* < .001, CFI = .91, TLI = 0.88, RMSEA = .086, 90% CI [.075, .098]. Reliability for the one factor (11 items) scale was good, α = .88.

#### XMQ discriminant validity

To assess discriminant validity of the XMQ, a mean score was computed for each participant by averaging the scores for the remaining 11 items (range: 1–5, M_XMQ_ = 2.88, SD_XMQ_ = 0.78), with 15.7 percent of participants scoring higher than one SD from the mean (> 3.67), and 3.8 percent of participants scoring higher than two SD from the mean (> 4.45). XMQ scores were subsequently correlated with participants’ self-reported screen time with smartphone, tablet, and computer, as well as other variables of interest (age and gender). The correlations between main study variables can be found in Table 2.

**Table 2:** Pearson’s correlations, means, and standard deviations of predictor variables.

	1	2^a^	3^a^	4^a^	5
1. Extended Mind Questionnaire	^-^	.47***	.10***	.13***	.24***
2. Smartphone Screen Time^a^		^-^	.11***	.09***	.24***
3. Tablet Screen Time^a^			-	.02	-.03
4. Computer Screen Time^a^				-	.08*
5. Age					-
*M*	31.74	2.70	0.85	1.64	14.15
*SD*	8.60	1.20	1.05	1.10	1.28

* *p* < .05

** *p* < .01

*** *p* < .001.

^a^ Means for smartphone (2), tablet (3), and computer (4) screen time are reported in hours.

As expected, XMQ scores were significantly and positively correlated with the amount of time participants spent on their smartphones, *r* = .47, *p* < .001, *R*^*2*^ = 0.22. XMQ scores were also positively correlated with tablet and computer use, although these relationships were considerably less strong, *r* = .10, *p* < .01, *R*^*2*^ = .01 and *r* = .13, *p* < .001, *R*^*2*^ = .02, respectively. The moderate to small correlations provide first indications that although related, extended mindedness is a different entity from screen time. Furthermore, this indicates that the respondents have a higher degree of extended mindedness towards the device most likely to be within constant reach, and which by extension has the highest probability of functioning as a person’s extension: the smartphone.

Results furthermore indicated a positive correlation between XMQ and age, with XMQ scores being higher for older participants, *r* = .24, *p* < .001, *R*^*2*^ = 0.06. Gender differences between XMQ scores and screen time were tested using t-tests. No gender differences were found on XMQ scores, *t*(934) = -1.07, *p* = .285. However, there were significant gender differences on screen time for smartphone, tablet and computer use. Female adolescents spent more time on their smartphone, *t*(921) = -8.31, *p* < .001, and tablet, *t*(945) = -3.32, *p* < .01, while males spent more time on the computer, *t*(918) = 2.94, *p* < .01. Thus, while genders differ on their amount of use per device, the degree of extended mind is equal between males and females.

#### XMQ criterion validity: Associations with XMQ and screen time

As a final step in demonstrating the construct validity of the XMQ, the predictive value of XMQ scores for a number of other measures (academic performance, sociability, and problematic media use) was assessed with a series of hierarchical regression models in which XMQ was added as a predictor above and beyond screen time on smartphone, age, and gender using the enter method. Smartphone screen time was chosen because it correlated most strongly with XMQ scores, and most aptly represents the idea of an extended mind device.

Previous research has operationalized extended mindedness as the amount of time spent on smartphones [1]. This research has found an association between usage time and a reduced inclination towards analytical reasoning, or cognitive miserliness. That is, cognitive misers tend to use smartphones as an external mind. Although this study found no relation of smart phone use with academic performance, other research indicates that the relation between smartphone and other digital media screen time and academic performance might be negative [41–43]. The assumption in these studies is that digital media use distracts students from their homework.

However, whereas in general purely spending much time on a digital screen may have a distracting effect, using the device for offloading information processes may also have positive consequences. For instance, getting information from the Internet or the memory banks of your phone may level the playing field between people with different cognitive skills levels [1, 13]. Moreover, outsourcing cognitive processing may free up memory capacity for creative problem solving [33]. Such positive effects of extended mindedness may cancel out negative ones. Therefore, if screen time and extended mindedness are indeed different entities, we may expect to find a negative effect of screen time on academic performance, and a smaller or no effect of XMQ.

In addition to academic performance, the current study also focuses on the relation between screen time and extended mindedness on sociability and problematic media use. Similar to our expectations regarding the relation with academic performance, we expect the degree of extended mindedness to be uniquely and distinctively related to these constructs alongside the amount of screen time.

For each dependent variable, a model including XMQ (step 2) was compared to the model without XMQ (step 1). The data met the assumption of independent errors (for all models, Durbin-Watson values were found between 1.769 and 2.073). Furthermore, multicollinearity was not a concern. Across all models for each predictor, VIF remained between 1.063 and 1.427. Regression results are presented for each dependent variable separately in Table 3.

**Table 3:** Hierarchical regression models of extended mind questionnaire.

		Academic Performance		Time Spent with Peers		Problematic Social Media Use		Problematic Internet Use	
		β	Δ*R*^2^	β	Δ*R*^2^	β	Δ*R*^2^	β	Δ*R*^2^
Step 1			.07***		.04***		.19***		.11***
	Gender	.12***		.01		12***		-.00	
	Age	-.18***		-.11**		.04		-.01	
	Smartphone Screen Time	-.13**		.16***		.15***		.08*	
Step 2			.00		.00		.18***		.21***
	Extended Mind	-.03		.06		.49***		.52***	
	Total *R*^2^		.07***		.04***		.37***		.31***

* *p* < .05

** *p* < .01

*** *p* < .001.

*Academic performance*. Compared to a model without XMQ, the full model was not found to explain significantly more variance in average grade (*R*^*2*^ = .07 for Step 1, Δ*R*^*2*^ = .001 for Step 2, *p* = .36, β_XMQ_ = -.03). In contrast, gender (β_*gender*_ = .12), age (β_*age*_ = -.18) and smartphone screen time (β_*time*_ = -.13) were significant predictors of (lower) average grade (all *p*’s < .01). This indicates that while screen time had a negative effect on academic performance, as found in prior research [1], the degree to which one uses the smartphone as an extension of the mind is not. The former two variables are therefore distinct from extended mindedness.

*Time spent with peers (TSWP)*. Compared to a model without XMQ, the full model was not found to explain significantly more variance in time spent with peers (R^2^ = .04 for Step 1, ΔR^2^ = .002 for Step 2, p = .15, β_XMQ_ = .06). While XMQ and gender (β_gender_ = .01) had no predictive effect on TSWP, age (β_age_ = -.11), and smartphone screen time (β_time_ = .16) did reveal to be significant predictors for TSWP (all p’s < .01).

*Problematic social media use (PSMU)*. XMQ was a significant predictor of PSMU above and beyond smartphone screen time, age, and gender (R^2^ = .19 for Step 1, ΔR^2^ = .18 for Step 2, p < .001, β_XMQ_ = .49). Gender (β_gender_ = .12) and screen time (β_time_ = .15) were also significant predictors for PSMU (both p’s < .001), whereas age was not.

*Problematic internet use (PIU*). XMQ was a significant predictor of PIU above and beyond screen time, age, and gender (R^2^ = .11 for Step 1, ΔR^2^ = .21 for Step 2, p < .001, β_XMQ_ = .52). Besides XMQ, only smartphone screen time was found to be a significant predictor of PIU (β_time_ = .08, p < .05), while age and gender had no significant predictive effect.

## Discussion

Today’s digital devices are becoming so thoroughly integrated in our lives, that they start taking over basic functions of the human mind. Especially *digital natives* are assumed to be at the forefront of these developments since they are the first generation to experience the full effect of technology on their social life, cognitive performance, and professional development. Despite the novelty of developments, much research effort has already been devoted to the absorption of digital mobile technologies into our daily lives. However, to date, an empirical measure of the idea of extended mindedness in relation to digital devices did not exist. The current paper represents a first attempt to provide a measure that can be used to study the impact of the absorption of mobile digital technology, represented by the degree of accessibility, endorsement, and reliance a person experiences towards digital media, in our cognitive and social processes.

The current paper presents two studies that provide strong indications that the Extended Mind Questionnaire (XMQ) is a highly reliable and valid measure for empirical research on the use and impact of digital technology. Factor analyses demonstrate good structural validity. Further tests indicate the one-dimensional 12-item XMQ scale is highly reliable, and has a high construct and criterion validity. As evidenced by, first, weak to moderate associations between extended mindedness and screen time, and, second, by the differential relationships of the two measures with other variables, the study shows that the measure of extended mindedness captures a novel dimension in our relation to technology. Third, further indications of construct validity are the theoretical plausibility of the ex post facto explanations of these relations, as we will discuss below. Taken together, the operationalization of the XMQ based on a theoretically sound conceptualization, combined with reliability and validity measures from the current studies, provide strong indications of the viability of the XMQ as a tool to measure extended mindedness.

The difference between the concept of extended mind and the more traditional measure of screen time is most clear in the finding that extended mindedness has no negative impact on academic performance, whereas a higher screen time does. The negative relation between screen time and average grade can be understood in terms of a time displacement effect. It has been argued that heavy usage of media can interfere with time spent for studying, reading or homework, in turn lowering academic achievement [41–44]. However, the degree of extended mindedness showed no association, suggesting that more reliance, accessibility and endorsement towards mobile devices may affect academic achievement in adolescence both negatively (through its connection with cognitive miserliness) and positively (by freeing up processing capacity). Disentangling these relative effects should be a prime challenge for future research.

A similar pattern of results was found when predicting the time spent with peers, although the relation between screen time and peers is positive. More screen time on a smartphone predicted more time spent with peers outside of school hours. Perhaps this is due to social networking possibilities of smartphone devices, or because adolescents who spent more time with their friends offline also put more time in socializing online with one another. The degree of extended mindedness did not show up as a significant predictor here, again illustrating the discriminant validity with screen time. A possible explanation for the different relationship of screen time and XMQ may be that adolescents with a high degree of extended mind use their device to socialize with peers, instead of meeting up outside of school. Thus, even if more smartphone use predicts more time spent with peers, those with a high degree of extended mind use their smartphone to connect and do not spent more time together *offline*. However, more research is needed to investigate this possible displacement effect of smartphones on time spent with peers offline.

Further indications for its validity and relevance for future research were obtained as the scale was used to predict two types of problematic Internet use. Both problematic social media and Internet use were positively predicted by both screen time and extended mindedness. However, a model with XMQ scores as an additional predictor above and beyond screen time was able to account for significantly more variance in problematic Internet and social media use. Moreover, inspection of the regression weights indicated that extended mind is a stronger predictor of problematic media use. These results suggest that XMQ is a better measure for detecting compulsive media use compared to simply using the amount of time spent online.

In all, our results indicate that the degree to which adolescents feel they use their devices as extended mind, has cognitive and behavioral consequences that are often different from the amount of time they spend on their screen.

### Prevalence of digital mind extension

One of the first issues in this domain would be to chart the prevalence of high extended mindedness in today’s public. In our sample of adolescents, only 3.8 percent of adolescents’ scores were above two standard deviations from the mean (above 4.45 on a 5-point scale). Compared to widespread academic and public discourse on excessive digital media use by adolescents, the prevalence of ‘extreme’ digital mind extension in our sample seems more modest–despite living in one of the world’s leading countries in terms of digital technology penetration [40] and having being involved with digital technology since an early age. It will be interesting for future research to see how these figures compare to other samples of age, education, and other social and psychological variables. Even more interesting will be to see how extended mindedness will develop as current generations grow older, and technologies progress.

The XMQ was developed and tested with young technology users in mind, as they are the generation that is most likely to be impacted the most by digital technology. However, given the formulation of its individual items, nothing would prevent the scale from being used to study extended mindedness in other age cohorts. In fact, the XMQ should be fully applicable in research on for instance age differences and technology use. Notably, it may be useful in providing input for the debate on ‘digital natives’. While it is undoubtedly true that the age cohort born after 1980 are the first to grow up in a digital landscape practically from birth, it is still contended whether this generation possesses unique and sophisticated skills in using these technologies, or whether they have radically different cognitive capacities and learning styles (e.g., [45–47]). The XMQ may aid future research in assessing whether different age groups have different approaches to digital technology as extensions of their minds.

As digital mobile consumer technology continues to develop at an astonishing rate, the impact of using digital technology as an extension of our minds in our daily lives is currently one of the major questions in science and society. Many of the hopes and anxieties surrounding digital technologies comes from the assumption that their capacity to become an integral part of the way humans use their minds makes these devices unique compared to earlier innovations. Though some fear that offloading cognitive tasks will lead to brain atrophy and dementia [48], others ]our digitally extended minds opening up extraordinary avenues of augmented intelligence (e.g., [49]). Where both parties agree on it seems, is that the current technology is bound to change the way we use our minds [3, 9, 28].

Our results show that using mobile and digital technology as part of your extended mind is not necessarily the same as using technology a lot. You can employ your phone to offload important brain functions, help in cognitive processes, or connect to your peers, which has distinct effects from simply spending much time on your phone. In contrast to measures used in earlier research, XMQ does not represent usage, but rather the degree of accessibility, reliance, and endorsement people experience with regard to their digital devices. Following the proposition by Clark and Chalmers [28], the degree to which one adheres to these three criteria defines the extent to which one uses a device as an extension of the mind. The one-dimensional factor structure of the XMQ suggests that the accessibility to, reliance on, and endorsement of technology are interdependent, and underlie a single construct. Thus, our findings underline that any investigation of effects of digital media use should go beyond screen time and focus more on the role that these digital media play in our lives. Furthermore, the concept of extended mindedness provides more neutral terms for the debate on technology to continue on. While XMQ was a significant predictor of problematic media use, the construct is not inherently posited as harmful. A moderate degree of digital extended mind may in fact be beneficial for many different cognitive and social tasks, provided we have reliable, trustworthy, and accessible devices.

Future research may use the XMQ to investigate the short and long-term impact of technology on such diverse matters as cognitive functioning, brain plasticity, social-emotional states, creativity, addiction, and health. Conversely, it can also be of use to study the antecedents of extended mindedness, such as psychological traits, social context (income, gender, parents, peers), and intelligence. We particularly encourage follow-up research using longitudinal or experimental research designs, as the current study is limited by its cross-sectional nature.

Whether the extension of our minds with digital technology will prove for better or for worse, in the debate on the impact of digital technology use on cognitive and socioemotional development we hope the XMQ will enable researchers to capture a novel and important dimension in our relationship to technology.

## Supporting information

S1 Table Original, 28-item version of the Extended Mind Questionnaire (XMQ) in Dutch. (DOCX)
