# Supplementary material for: Has your smartphone replaced your brain? Construction and validation of the Extended Mind Questionnaire (XMQ)
Source: PLoS One. 2018 Aug 31;13(8):e0202188. doi: 10.1371/journal.pone.0202188 (PMC6118357; doi:10.1371/journal.pone.0202188)
Supplement: S1 Table — (DOCX) [file pone.0202188.s001.docx]

S1 Table. Original, 28-item version of the Extended Mind Questionnaire (XMQ) in Dutch

| **Item** | Item wording |
| --- | --- |
| **1** | Ik vind het lastig om online informatie op te zoeken |
| **2** | Wanneer ik informatie wil opzoeken, gebruik ik automatisch het internet |
| **3** | Online informatie is voor mij op ieder moment beschikbaar |
| **4** | Mijn smartphone, tablet of computer werkt altijd precies zoals ik hem nodig heb |
| **5** | Wanneer ik me iets niet kan herinneren, gebruik ik automatisch mijn smartphone, tablet of computer om het op te zoeken |
| **6** | Als ik contact wil maken met vrienden pak ik meteen mijn smartphone, tablet of computer |
| **7** | Ik vind het logischer om iemand in het echt aan te spreken dan via sociale media |
| **8** | Ik heb mijn smartphone, tablet of computer altijd binnen handbereik |
| **9** | Op sociale media zijn mijn vrienden op ieder moment beschikbaar |
| **10** | Voor school/studie/werk kan ik niet zonder mijn smartphone, tablet of computer |
| **11** | Bij bijna alles wat ik onderneem, gebruik ik mijn smartphone, tablet of computer |
| **12** | Als ik iets te weten wil komen, kan ik dat heel goed zonder het internet |
| **13** | Sociale media zijn voor mij belangrijk in het contact met mijn vrienden |
| **14** | Ik kan niet zonder sociale media bij het onderhouden van mijn vriendschappen |
| **15** | Als ik ergens niet op kan komen, grijp ik meteen naar mijn smartphone, tablet of computer om het op te zoeken |
| **16** | Als ik iets echt moet onthouden, sla ik het op in mijn smartphone, tablet of computer |
| **17** | Ik ben afhankelijk van mijn smartphone, tablet of computer voor het onthouden van belangrijke dingen zoals verjaardagen en afspraken |
| **18** | Ik kan prima zonder mijn smartphone, tablet of computer |
| **19** | Ik ben erg afhankelijk van mijn smartphone, tablet of computer |
| **20** | Als ik iets niet weet gebruik ik mijn smartphone, tablet of computer |
| **21** | Ik vertrouw informatie uit mijn smartphone, tablet of computer net zoveel als mijn eigen geheugen |
| **22** | Als ik iets op internet zie, neem ik het niet automatisch voor waar aan |
| **23** | Wat ik op sociale media voorbij zie komen, geloof ik alsof ik er zelf bij ben geweest |
| **24** | Ik vertrouw sociale media minder dan contact in het echt |
| **25** | Mijn smartphone, tablet of computer vergist zich nooit |
| **26** | Mijn smartphone, tablet, of computer is een onderdeel van mezelf |
| **27** | Ik gebruik mijn smartphone, tablet, of computer als verlengstuk van mezelf |
| **28** | Mijn smartphone, tablet of computer werkt vaak als mijn geheugen |
